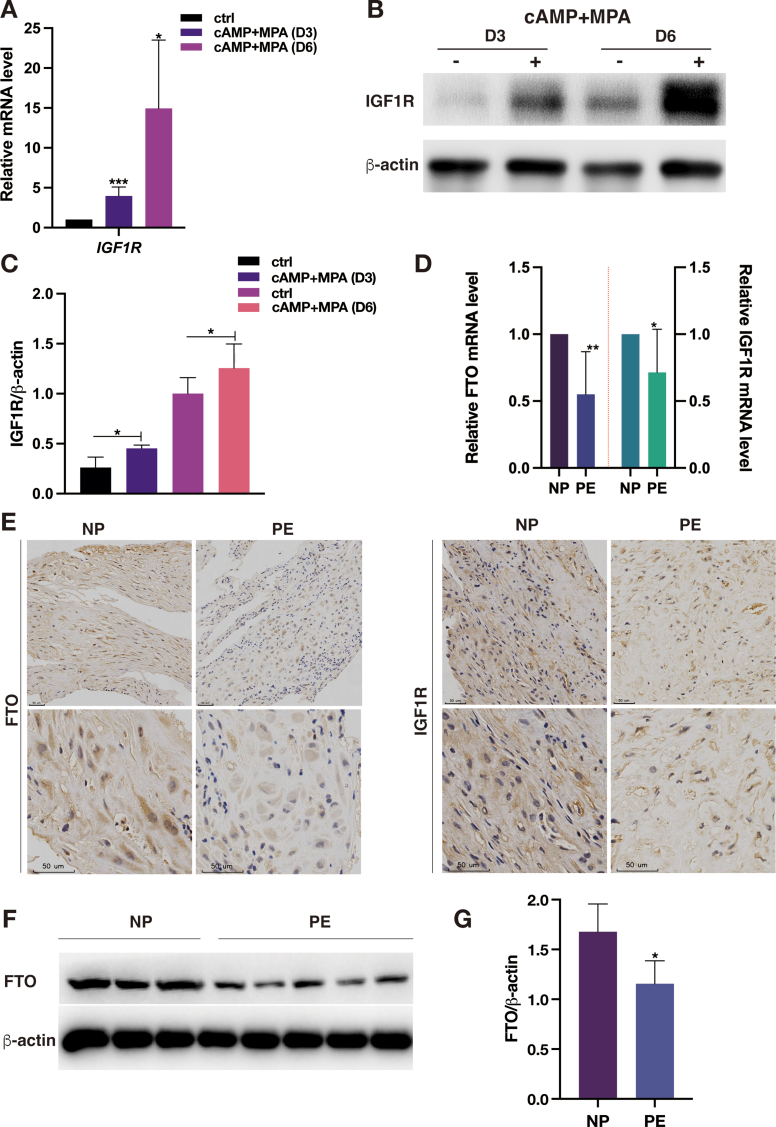# Exploring the role of FTO in preeclampsia pathogenesis: Insights into m^6^A modification and decidualization

**DOI:** 10.1016/j.gendis.2024.101504

**Published:** 2024-12-24

**Authors:** Jing Tong, Liang Zhang, Jing Bai, Cong Zhang

**Affiliations:** a Center for Reproductive Medicine, Ren Ji Hospital, Shanghai Jiao Tong University School of Medicine, Shanghai 200135, China; b Shanghai Key Laboratory for Assisted Reproduction and Reproductive Genetics, Shanghai 200135, China; c Research Center of Translational Medicine, Jinan Central Hospital Affiliated to Shandong First Medical University, Jinan, Shandong 250013, China; d Jinan Maternal and Child Health Care Hospital Affiliated to Shandong First Medical University, Jinan, Shandong 250001, China; e Shandong Provincial Key Laboratory of Animal Resistance Biology, College of Life Sciences, Shandong Normal University, Jinan, Shandong 250014, China; f Shandong Provincial Key Laboratory of Reproductive Medicine, Jinan, Shandong 250001, China

Preeclampsia (PE) poses a grave threat to both maternal and fetal health, yet its intricate cellular and molecular mechanisms remain shrouded in mystery.^1^ Recent research has increasingly focused on the role of maternal-impaired decidualization in the development of PE.^2^ In this context, the obesity-associated protein FTO has emerged as a significant factor, acting as a demethylase for N6-methyladenosine (m^6^A) modification. Our study aimed to unravel how FTO enhanced glycolysis and vascular formation during decidualization. Mechanistically, FTO reduced the m^6^A methylation of IGF1R (insulin-like growth factor 1 receptor) transcripts, thereby stabilizing IGF1R and augmenting its expression through a YTHDF2 (YTH m^6^A RNA binding protein F2)-dependent pathway, an essential process for stromal cell decidualization. Additionally, IGF1R was required for regulating COX2 (cytochrome C oxidase assembly factor) and VEGFA (vascular endothelial growth factor A) expression, while also modulating AKT (protein kinase B) activity during decidualization. Intriguingly, we unearthed that *FTO* and *IGF1R* expression in the decidua were negatively correlated with systolic blood pressure. In summary, our study uncovers a novel mechanism involving FTO-mediated m^6^A demethylation orchestrating *IGF1R* mRNA modification through YTHDF2, thereby offering promising avenues for targeted FTO modulation in the treatment of hypertensive disorders in PE, ultimately translating to improved outcomes for both mothers and fetuses.

We initiated decidualization by stimulating with cyclic adenosine monophosphate (cAMP, 0.5 mmol/L) and medroxyprogesterone acetate (MPA, 1 μmol/L). Following decidualization periods of three and six days, we noted a notable elevation in the mRNA levels of key decidualization markers, notably *PRL* (prolactin) and *IGFBP1* (insulin-like growth factor binding protein 1), within the human endometrial stromal cells (HESCs). Remarkably, in parallel with the decidualization progression, we detected a substantial surge in *FTO* mRNA expression in the induced HESCs compared with their control counterparts (Fig. S1A). This effect was further confirmed through Western blot analysis, which showed a corresponding rise in FTO protein levels after decidualization (Fig. S1B, C). Further investigation of global m^6^A levels in decidualized HESCs revealed a significant decrease during decidualization (Fig. S1D). This intriguing trend was also validated through m^6^A dot blot assays, confirming the decreased m^6^A content in mRNA during decidualization (Fig. S1E). Additionally, we observed a significant increase in the formation of tube-like structures in the conditioned culture media (Fig. S2A, B). Moreover, there was a notable increase in glucose uptake (Fig. S2C) and lactate levels (Fig. S2D) in the culture media of decidualized HESCs compared with control cells.

To evaluate the functional impact of FTO, we utilized siRNAs to suppress *FTO* expression. Strikingly, reducing FTO expression in HESCs led to a marked inhibition of *PRL* and *IGFBP1* expression (Fig. S2E). Simultaneously, we observed significantly increased global m^6^A levels within mRNA during decidualization. This distinct alteration was consistently detected using the m^6^A RNA methylation quantification kit and the m^6^A dot blot assay (Fig. S2F, G).

To gain a deeper understanding of FTO's functions, valuable resources including data from the m6Avar database (www.m6avar.renlab.org) and insights from the UCSC Genome Browser (http://genome.ucsc.edu) were utilized. Upon identifying IGF1R as a potential direct target of FTO, we embarked on an exploration of the regulatory mechanism governing IGF1R expression through FTO, with particular emphasis on its interplay with YTHDF2 binding. Impressively, we observed a significant reduction in *IGF1R* mRNA expression in HESCs with suppressed FTO (Fig. S3A). Furthermore, the levels of IGF1R protein expression followed a similar pattern, exhibiting a positive correlation with FTO expression in HESCs (Fig. S3B, C). Additionally, through RNA immunoprecipitation-quantitative PCR assays using an anti-FTO antibody, we successfully demonstrated a direct binding between *FTO* and *IGF1R* mRNA in HESCs (Fig. S3D). Similarly, when we utilized an anti-YTHDF2 antibody in RNA immunoprecipitation-quantitative PCR assays, we observed a significant decrease in the binding affinity of YTHDF2 towards *IGF1R* mRNA in FTO-silenced HESCs (Fig. S3E). Moreover, experiments involving actinomycin D showed that the RNA decay rates of *IGF1R* transcripts were accelerated upon *FTO* knockdown compared with control cells (Fig. S3F). These observations collectively suggest that FTO, through its influence on m^6^A modification, mediates the process of *IGF1R* mRNA decay, facilitated by YTHDF2 (Fig. 1A).

**Figure 1:**
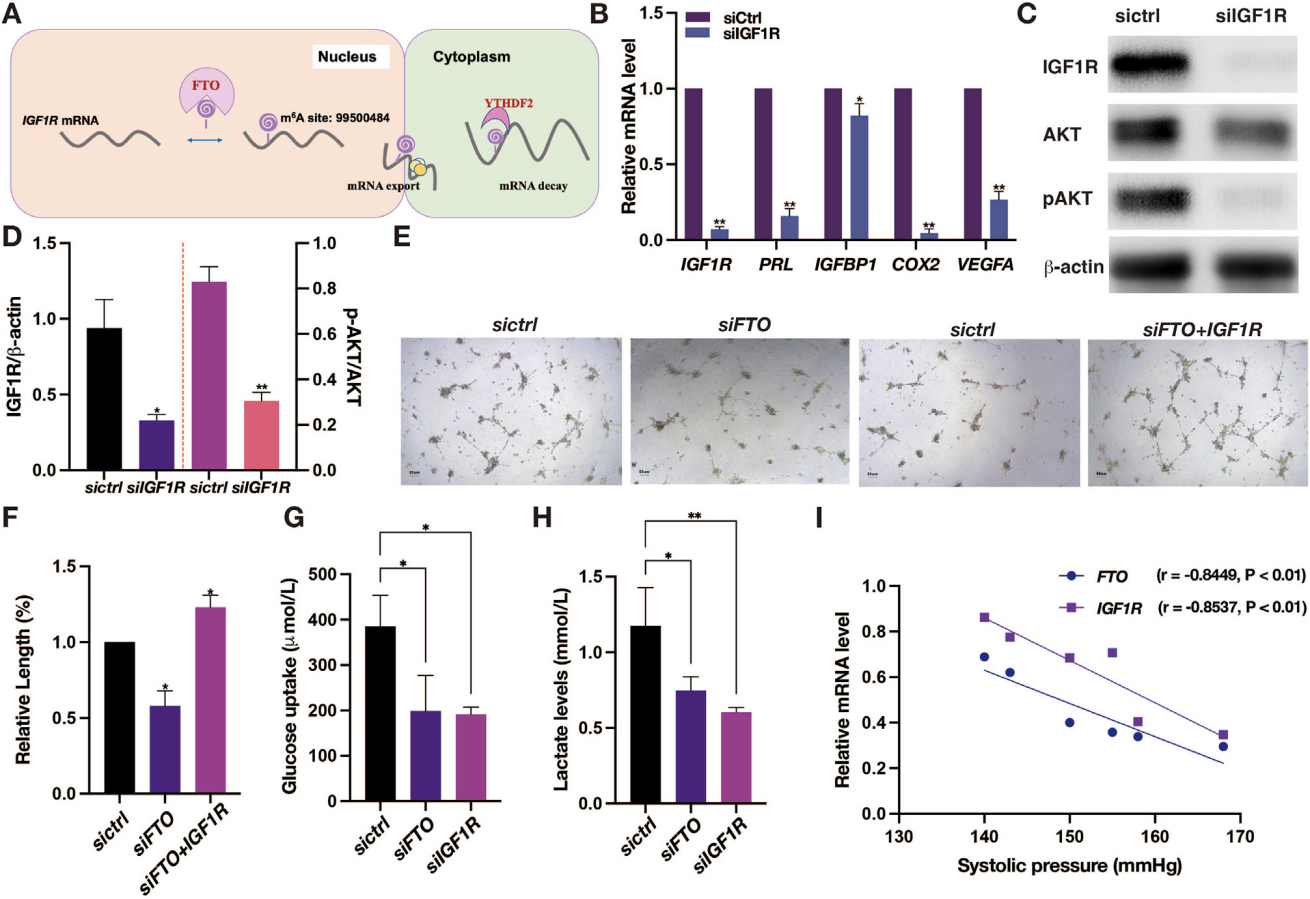
FTO's key role in preeclampsia pathogenesis. **(A)** Schematic representation of the regulatory mechanism of FTO on IGF1R expression contingent upon YTHDF2 binding. **(B)** Comparative mRNA expression levels of *IGF1R*, *PRL*, *IGFBP1*, *COX2*, and *VEGFA*, normalized to *ACTB*, following *IGF1R* knockdown in HESCs during decidualization. **(C, D)** Western blot analysis showed the protein expression of IGF1R, AKT, and p-AKT in decidualized HESCs, with beta-actin serving as a loading control. **(E)** Visual representation of morphological changes in tube formation by HUVECs treated with conditioned medium obtained from decidualized HESCs transfected with control siRNA or siRNAs targeting *FTO* and *siIGF1R*. **(F)** Quantitative image analysis revealed the skeleton length of angiogenic structures (*n* = 3, assessed using the nonparametric Mann–Whitney test). **(G)** Reduced glucose uptake was observed upon *FTO* and *IGF1R* knockdown in HESCs during decidualization. **(H)** Suppressed lactate production resulting from *FTO* and *IGF1R* knockdown in HESCs during decidualization. **(I)** Pearson's correlation analysis showcased the relationship between systolic blood pressure in preeclampsia patients and the relative mRNA expression levels of *FTO* and *IGF1R*. All data were represented as mean ± standard deviation. Statistically significant differences are denoted by asterisks (∗*P* < 0.05, ∗∗*P* < 0.01, ∗∗∗*P* < 0.001). Scale bars = 50 μm. IGF1R, insulin-like growth factor 1 receptor; YTHDF2, YTH m^6^A RNA binding protein F2; PRL, prolactin; IGFBP1, insulin-like growth factor binding protein 1; COX2, cytochrome C oxidase assembly factor; VEGFA, vascular endothelial growth factor A; ACTB, actin beta; HESCs, human endometrial stromal cells; AKT, protein kinase B; p-AKT, phosphorylated AKT.

Subsequently, we investigated the role of IGF1R in decidualization. In line with the progression of decidualization, the expression of *IGF1R* mRNA significantly increased in the induced HESCs compared with their control counterparts (Fig. S4A). This finding was further supported by Western blot analysis, which showed a corresponding increase in IGF1R protein levels after decidualization (Fig. S4B, C). Notably, the reduction of *IGF1R* expression in HESCs significantly inhibited the expression of *PRL*, *IGFBP1*, *COX2*, and *VEGFA* (Fig. 1B). Additionally, we analyzed protein lysates to assess the expression of IGF1R, total AKT, phosphorylated AKT, and actin as a loading control. In HESCs transfected with *IGF1R siRNA*, both the total and phosphorylated states of AKT exhibited a marked decrease compared with the controls after decidualization (Fig. 1C, D).

Expanding on the findings, we conducted tube formation assays, glucose uptake assays, and lactate level assays to further elucidate the functions of FTO and IGF1R. Particularly noteworthy, the reduced FTO expression in HESCs resulted in a significantly inhibited tube formation in the conditioned culture media (Fig. 1E, F). Additionally, there was a decrease in the capability of glucose uptake (Fig. 1G) and lactate levels (Fig. 1H) in the culture media of decidualized HESCs, relative to those observed in the culture media of control cells. Interestingly, the overexpression of *IGF1R* partially reversed the inhibitory effect of *FTO siRNA* on tube formation (Fig. 1E, F).

Returning to our clinical data, we further explored changes in decidual FTO and IGF1R expression in patients with PE. The clinical information of 32 participants is presented in Table S2. We observed a significant reduction in the mRNA levels of *FTO* and *IGF1R* in the PE group compared with the control group (Fig. S4D). Immunohistochemistry on paraffin sections obtained from decidual tissue samples of PE patients and normal pregnant individuals revealed predominant localization of FTO and IGF1R protein in the cytoplasm of decidual cells. Importantly, we observed a noticeable decrease in FTO and IGF1R protein levels in the PE group compared with the control group (Fig. S4E). A corresponding decrease in FTO protein levels was evident in the PE group compared with the control group (Fig. S4F). Subsequently, we conducted an analysis of Pearson's correlation, which unveiled a negative correlation between the relative mRNA expression levels of both *FTO* (*r* = −0.8449, *P* < 0.01) and *IGF1R* (*r* = −0.8537, *P* < 0.01) with systolic blood pressure (Fig. 1I). These compelling findings highlight the pivotal roles of both FTO and IGF1R in the clinical manifestations of PE, further emphasizing their significance in this context.

Glycolysis is vital for human and mouse decidualization, with its disruption playing a critical role in human decidualization and PE onset.^3^^,^^4^ Angiogenesis is required for embryo implantation and early post-implantation decidual angiogenesis. Reduced COX2 expression in the decidua of PE individuals affects PE via VEGFA.^5^ In conclusion, our findings illuminate the intricate interplay between FTO and m^6^A demethylation, offering the tantalizing prospect of m^6^A methylation control as a potential therapeutic strategy for PE.

## Ethics declaration

The ethical aspects of this study were reviewed and approved by the Renji Hospital Ethics Committee, Shanghai Jiaotong University School of Medicine (No: 2017041412, 13 April 2017). All patients provided written informed consent.

## Funding

This study was supported by the 10.13039/501100012166National Key R&D Program of China (No. 2019YFA0802600) and the 10.13039/501100001809National Natural Science Foundation of China (No. 32170863, 31871512) to Cong Zhang. Support was also received from grants from the Shanghai Commission of Science and Technology (China) (No. 20DZ2270900) and the Open Project of Shandong Provincial Key Laboratory of Reproductive Medicine (China) (No. SDKL2017018).

## CRediT authorship contribution statement

**Jing Tong:** Data curation, Investigation, Methodology, Visualization, Writing – original draft, Writing – review & editing. **Liang Zhang:** Methodology, Software, Visualization. **Jing Bai:** Data curation, Investigation, Writing – original draft. **Cong Zhang:** Conceptualization, Funding acquisition, Methodology, Project administration, Writing – review & editing.

## Conflict of interests

The authors declared no competing interests.

## Figures and Tables

**figs1:**
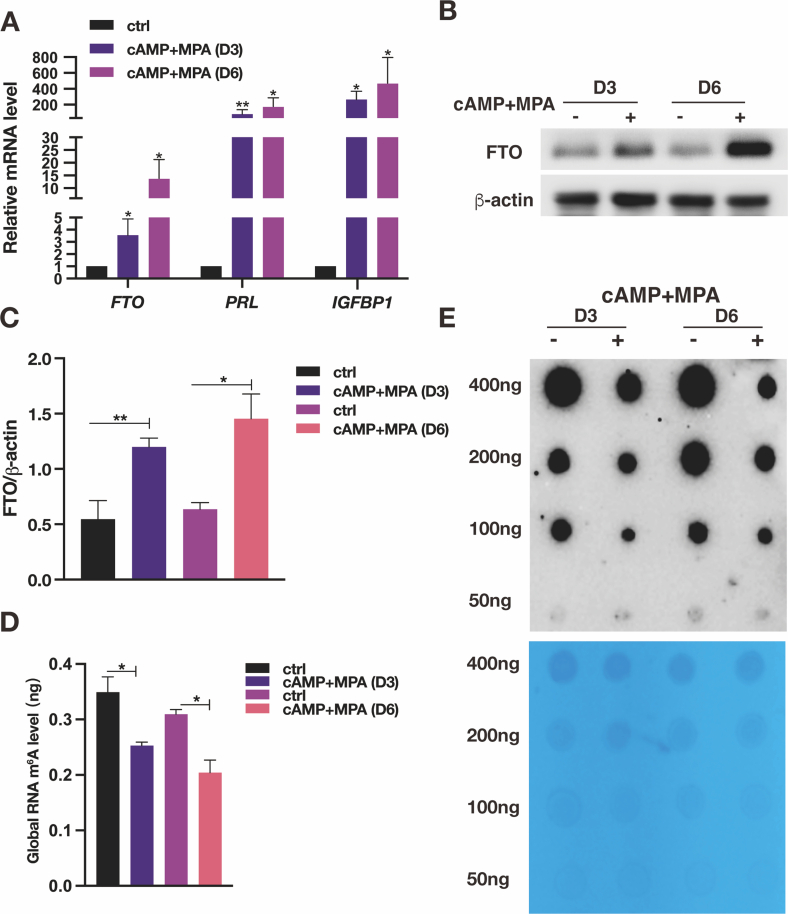


**figs2:**
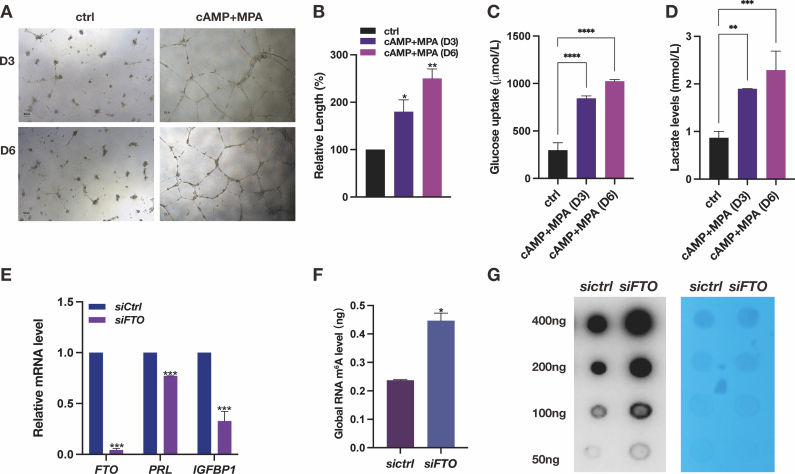


**figs3:**
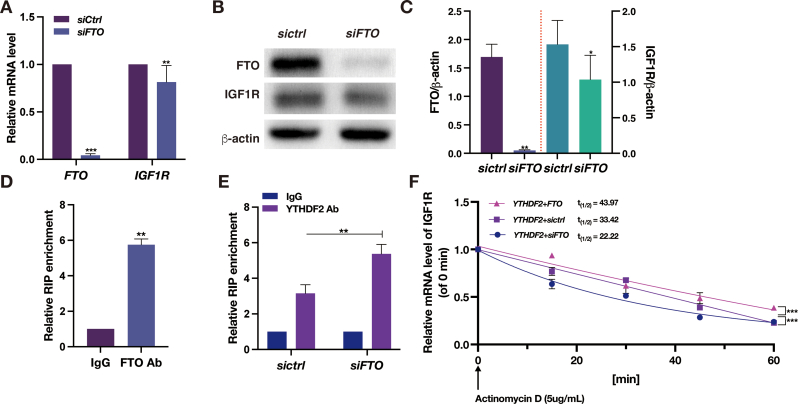


**figs4:**