# Supplementary material for: Exploring the role of FTO in preeclampsia pathogenesis: Insights into m6A modification and decidualization
Source: Genes Dis. 2024 Dec 24;12(4):101504. doi: 10.1016/j.gendis.2024.101504 (PMC11960631; doi:10.1016/j.gendis.2024.101504)
Supplement: Multimedia component 1 [file mmc1.docx]

**Exploring the Role of FTO in Preeclampsia Pathogenesis: Insights into m^6^A Modification and Decidualization**

**Methods and Materials**

***Participants and sample collection***

A total of 32 participants were enrolled in this study, which was conducted at the Obstetrics and Gynecology Department of Ren Ji Hospital, School of Medicine, Shanghai Jiao Tong University, between April 2015 and June 2019. The study population consisted of 16 pregnancies complicated by PE, while 16 women with normal pregnancies (NP) were included as the control group.

PE was diagnosed based on the criteria established by the International Society for the Study of Hypertension in Pregnancy ^15^. It was defined as the presence of new-onset hypertension and proteinuria or other end-organ damage occurring after 20 weeks of gestation. Participants with chronic hypertension, diabetes, renal disease, thyroid disease, fetal anomalies, multiple pregnancies, or other pregnancy-related complications were excluded from the study.

Ethical approval for this study was obtained from the Ethics Committee of Ren Ji Hospital, and informed consent was obtained from all participants prior to their inclusion. All pregnancies were delivered via cesarean section. Immediately following delivery, samples of the decidua basalis were collected from the gauze used to scrub the uterus. To ensure the removal of any blood, the tissue was rinsed with sterilized saline. Subsequently, the collected tissue was snap frozen in liquid nitrogen and stored at -80 °C until further analysis.

***Decidualization induction and cell transfection***

The immortalized HESCs were generously provided by Dr. Haibin Wang from Xiamen University, Xiamen, China. To induce decidualization *in vitro*, HESCs were cultured in DMEM/F12 medium supplemented with 2% charcoal-stripped fetal bovine serum (Biological Industries, Beit Haemek, Israel), 0.5 mmol/L double cyclic adenosine monophosphate (cAMP; Sigma-Aldrich, St. Louis, MO USA), and 1 μmol/L medroxyprogesterone-17-acetate (MPA; Sigma-Aldrich) for three or six days. The culture medium was refreshed every other day to maintain the induction environment.

Before transfection, HESCs (2 × 10^5^ cells) were seeded into 6-well plates and allowed to attach for one day. Subsequently, transfection of *siRNAs* and/or plasmid DNA was performed using Lipofectamine 3000 Reagent (Invitrogen, Carlsbad, CA, USA) following the manufacturer's protocol. The *siRNAs* used in this study were purchased from Biotend (Biotend Biotechnology Co., Shanghai, China), and their corresponding sequences are provided in Table S1. *IGF1R* cDNA was synthesized and inserted into pcDNA3 (Thermo Fisher Scientific, Cleveland, OH, USA). Regarding to decidualization after transfection, the cells were induced to decidualization by incubation with cAMP and MPA for four days after transfection.

***Quantitative PCR (qPCR) and Western blot analysis***

Total RNA was extracted from cultured cells or tissue samples using the Foregene total RNA isolation kit (Foregene Co., Chengdu, Sichuan, China) according to the manufacturer's instructions. Subsequently, cDNA was synthesized using the PrimeScript RT Master Mix Perfect Real Time Kit (Takara Biotechnology Co., Dalian, China). qPCR was performed to measure the expression of target genes using SYBR-Green Dye and specific primers provided in Table 1. *ACTB* was used as the internal control, and all reactions were performed in triplicate to ensure robustness. Data analysis was conducted using the 2^-ΔΔCt^ method with a calibrator sample for relative quantification.

Protein extraction from cellular or tissue specimens was executed utilizing the following methodology. The cells were collected and subjected to lysis in an ice-cold radioimmunoprecipitation assay (RIPA) lysis buffer (Beyotime, Shanghai, China), supplemented with a protease inhibitor (Roche, Penzberg, Germany) as well as a phosphatase inhibitor (Roche). The ensuing primary antibodies were leveraged for the purpose of Western blot analysis: recombinant anti-FTO antibody (Abcam Co., Shanghai, China), IGF1 receptor β antibody (Cell Signaling Technology (CST), Boston, MA, USA), AKT antibody (CST), phospho-Akt (Ser473) (CST), and beta-actin (1:5000 dilution, Santa Cruz Biotechnology, Santa Cruz, CA, USA). Post incubation with the aforementioned primary antibodies, the specimens were further subjected to a 60-min incubation at ambient temperature with a goat anti-rabbit peroxidase-conjugated secondary antibody (1:5000 dilution, Proteintech, Wuhan, Hubei, China). A chemiluminescence reagent (Thermo Fisher Scientific) was employed for blot visualization.

***Immunohistochemistry***

Tissue sections of 4 µm thickness were prepared from paraffin-embedded decidual tissue obtained from both the PE and NP cohorts. These sections were initially fixed in a 4% paraformaldehyde solution, then subjected to a series of treatments involving alcohol and dimethylbenzene. Following this, the sections were incubated in citric acid antigen retrieval buffer and subsequently heated in a microwave oven. After heating, the sections were rinsed thrice with phosphate-buffered saline (PBS). This was followed by immersing the slides in 3% hydrogen peroxide solution for a period of 30 min, effectively blocking any endogenous peroxidase activity. The sections were then treated with 3% bovine serum albumin (BSA) for a 30-min duration at room temperature, to ensure proper blocking.

After blocking, the tissue sections were then placed in a wet box and incubated overnight at 4°C with the FTO antibody (1:500 dilution, Abcam Co.) and IGF1 receptor β antibody (1:500 dilution, CST). The sections were then rinsed thrice with PBS and incubated with a goat anti-rabbit secondary antibody (1:1 dilution, DAKO, Denmark) for 1 h at ambient temperature. The peroxidase substrate solution from the diaminobenzidine kit (DAKO) was used to instigate the coloration reactions. The slides were then counterstained with hematoxylin, dehydrated using 95% alcohol, and ultimately mounted in neutral balsam. Photographic documentation was facilitated using a microscope (Zeiss, Germany). To validate the specificity of the immunohistochemical staining, control sections were treated with pre-immune serum in lieu of the primary antibody.

***m^6^A quantification and dot blot analysis***

The alterations in global m^6^A levels within mRNA were assessed using the EpiQuik m^6^A RNA Methylation Quantification Kit (Colorimetric, Epigentek, Farmingdale, NY, USA) following the standard manufacturer's guidelines. Each reaction utilized an optimal RNA amount of 200 ng. Initially, the binding solution and sample RNA were added into individual wells to facilitate RNA binding. After thorough washing, capture antibodies were introduced to each well to capture the m^6^A-modified RNA. Subsequently, color developing solution was applied to induce color development, and the absorbance at 450 nm was measured using a microplate reader within a time frame of 2 to 15 min.

According to a published protocol ^16^, the dot blot analysis procedure is delineated as follows: Initiate by isolating and quantifying total RNA from cells utilizing TRIzol™ reagent (Thermo Fisher Scientific), adhering to the manufacturer's recommendations. Ensure the nitrocellulose membrane is firmly set within the Dot Blot apparatus and verify its integrity by testing for leakages with a droplet of PBS containing bromophenol blue. Allocate 1/5th of the eluted m^6^A RNA to the apparatus in designated concentrations, followed by a crosslinking process using a UV cross-linker. After this, cleanse the membrane with a wash buffer to eradicate any unattached RNA, and precondition it with a blocking buffer at ambient temperature. Thereafter, submerge it in a mixture featuring the Anti-m^6^A antibody at a 1:500 dilution (Abcam Co.). Post several wash cycles, administer the SuperSignal™ Western blotting reagent (Thermo Fisher Scientific) to the membrane and position it against an autoradiography film in a light-shielded setting. As a final step, process the film to interpret the outcomes.

***RNA immunoprecipitation (RIP) and RNA stability analysis***

The RIP assays were performed utilizing the Magna RIP Kit (Millipore, New Bedford, MA, USA) in strict adherence to the manufacturer's guidelines. Cells underwent preparation with RIP lysis buffer, and RNA-protein complexes were subsequently immunoprecipitated with the anti-FTO antibody (Proteintech, Rosemont, IL, USA) and anti-YTHDF2 antibody (CST) as well as normal rabbit IgG. Afterward, the co-precipitated RNAs were isolated using a phenol: chloroform: isoamyl alcohol mixture and advanced to qPCR analysis. For reference, a control amplification was executed on the input RNA prior to immunoprecipitation.

In order to assess the stability of *IGF1R*, cells were treated with Actinomycin D (5 mg/ml, CST) to halt transcription. Collections were made at intervals of 0 min, 20 min, 40 min, and 60 min following this termination of transcription. The remaining levels of *IGF1R* were then determined through RNA extraction and quantified using qPCR.

***Tubal formation analysis***

The tube formation assay involves loading a pre-chilled 24-well plate with Cultrex UltiMatrix Reduced Growth Factor Basement Membrane Extracts (Biotech Co., Shanghai, China) and solidifying it. Once the gel is set, a mixture of conditioned media and resuspended HEVUCs (Thermo Fisher Scientific) is added to the wells. The formation of tubes Tube formation's time and duration depend on the conditioned media's angiogenic factor concentration, with optimal results observed between 3 to 12 h. Following peak formation, the media is carefully aspirated, and the wells are washed. The tubes can then be visualized immediately or fixed for later observation. To quantify the tube networks, various parameters such as tube count, branch sites, and tube lengths are measured using Image J Version 1.49 software (National Institutes of Health) ^17^.

***Glucose uptake and lactate production assay***

The Glucose Uptake Colorimetric Assay Kit from Abcam was employed to assess glucose uptake as per the manufacturer's guidelines. HESCs at a concentration of 1x10^4^ were transferred to 96-well culture plates and incubated at 37℃ overnight. On the subsequent day, the cells underwent a 2-h glucose starvation. A 40 -min incubation with 100μl of Krebs-Ringer-Phosphate-HEPES was followed by a 20 min incubation post-injection of 10μl of 10mM 2-DG per well. The cells were then processed with extraction buffer to determine glucose uptake, which was quantified by measuring the optical density (OD) at 412 nm. In a parallel assay, the L-Lactate Assay Kit (Colorimetric) from Abcam was used to gauge lactate production. HESCs were deposited into 96-well plates and incubated at 37℃ overnight. After a 2-h starvation period, the supernatant was collected, and the lactate levels were determined using a microplate reader at 450 nm.

***Statistical analysis***

Data are presented as mean ± standard deviation. The Student's *t*-test or nonparametric Mann-Whitney test was utilized to determine differences between two groups, while ANOVA was employed for multiple group comparisons. The correlation between *FTO* and *IGF1R* expression levels and systolic pressure characteristics was examined using Pearson’s *χ^2^* test. All statistical procedures were conducted using SPSS software version 20.0 (SPSS, Inc., Chicago, IL, USA) and GraphPad Prism 7.0 (GraphPad Software, La Jolla, CA, USA). Each experiment was replicated at least three times independently for data collection. Significance levels were denoted as: * *P* < 0.05, ** *P* < 0.01, ****P* < 0.001, and **** *P* < 0.0001.

**Figure legends**

**Figure S1. Expression and Methylation Changes During HESC Decidualization. (A)** Expression profiles of *FTO*, prolactin (*PRL*), and insulin-like growth factor binding protein (*IGFBP1*) in human endometrial stromal cells (HESCs) during decidualization induced by cAMP and MPA. **(B and C)** Protein expression of FTO in decidualized HESCs, with beta-actin as a loading control. **(D)** Reduction in m^6^A mRNA methylation level in HESCs during decidualization induced by cAMP and MPA. **(E)** Global m^6^A abundance in mRNA from decidualizing HESCs measured using the m^6^A dot blot assay, with methylene blue staining as a loading control. All data are presented as mean values with standard deviation (SD). Statistical significance differences are indicated by asterisks (**P*<0.05, ***P*<0.01, ****P*<0.001).

**Figure S2. Functional and Molecular Changes During HESC Decidualization. (A)** Visualization of tube formation morphology in HUVECs treated with conditioned medium from decidualized HESCs induced by cAMP and MPA. **(B)** Quantitative image analysis displaying the skeleton length of angiogenic structures (*n*=3, assessed using the nonparametric Mann-Whitney test). **(C)** Enhanced glucose uptake observed in HESCs undergoing decidualization. **(D)** Elevated lactate production in HESCs during decidualization. **(E)** Relative mRNA expression of *FTO*, *PRL* (prolactin), and *IGFBP1* (insulin-like growth factor binding protein 1) normalized to ACTB following *FTO* knockdown in HESCs during decidualization induced by cAMP and MPA. **(F)** Increase in m^6^A mRNA methylation levels in HESCs undergoing decidualization following *FTO* knockdown. **(G)** Global m^6^A abundance in mRNA during decidualization induced by cAMP and MPA, and in *FTO* knockdown cells, measured by the m^6^A dot blot assay, with methylene blue staining as a loading control. All data are presented as mean values with standard deviation (SD). Statistical significance differences are indicated by asterisks (**P*<0.05, ***P*<0.01, ****P*<0.001). Scale bars correspond to 50 μm.

**Figure S3. Molecular insights into FTO’s impact on IGF1R Expression. (A)** Relative mRNA expression of *IGF1R* in HESCs following *FTO* knockdown. **(B and C)** Protein expression levels of FTO and IGF1R in HESCs after *FTO* knockdown, with beta-actin as a loading control. **(D)**  qPCR analysis of RNA immunoprecipitation (RIP) assays, revealing direct binding between the FTO protein and IGF1R (*n*=3, assessed using the nonparametric Mann-Whitney test). **(E)** qPCR analysis of RIP assays showing direct binding between the YTHDF2 protein with *IGF1R* (*n*=3, assessed using the nonparametric Mann-Whitney test). **(F)** Measurement of *IGF1R* mRNA half-life (t_1/2_) by qPCR in 293T cells transfected with control *siRNA* or *siFTO* (*n*=3, assessed using the nonparametric Mann-Whitney test). All data are presented as mean values with standard deviation (SD). Statistically significant differences are denoted by asterisks (**P*<0.05, ***P*<0.01, ****P*<0.001).

**Figure S4. Expression and Localization of IGF1R and FTO in Decidual Tissues. (A)** Assessment of *IGF1R* expression in human endometrial stromal cells (HESCs) undergoing decidualization induced by cAMP and MPA. **(B and C)** The protein expression of IGF1R in decidualized HESCs, with beta-actin serving as a loading control. **(D)** Relative mRNA expression of *FTO* and *IGF1R* normalized to *ACTB*. **(E)** Representative immunohistochemistry staining of FTO and IGF1R in decidua sections from both preeclampsia (PE) and normal pregnancy (NP) samples. **(F and G)** Protein expression of FTO in PE and NP decidua, with beta-actin as a loading control. All data are presented as mean values with standard deviation (SD). Statistically significant differences are indicated by asterisks (**P*<0.05, ***P*<0.01, ****P*<0.001). Scale bars correspond to 50 μm.
