# Supplementary material for: Exploring the role of FTO in preeclampsia pathogenesis: Insights into m6A modification and decidualization
Source: Genes Dis. 2024 Dec 24;12(4):101504. doi: 10.1016/j.gendis.2024.101504 (PMC11960631; doi:10.1016/j.gendis.2024.101504)
Supplement: Multimedia component 2 [file mmc2.docx]

**Table S1. The sequences of siRNAs and primers used in this study.**

| **Sequences of siRNAs used in this study.** | | |
| --- | --- | --- |
| gene | sense | antisense |
| FTO | GUGGCAGUGUACAGUUAUATT | UAUAACUGUACACUGCCACTT |
| IGF1R | GCUGGAAACUCUUCUACAATT | UUGUAGAAGAGUUUCCAGCTT |
| **Sequence of primers used for real-time PCR** | | |
| Gene | Primer (Forward) | Primer (Reverse） |
| ACTB | GGGAAATCGTGCGTGACATTAAG | TGTGTTGGCGTACAGGTCTTTG |
| PRL | CATATTGCGATCCTGGAATGAG | GATGAACCTGGCTGACTATCA |
| IGFBP1 | GGCACAGGAGACATCAGGAGAA | GGTAGACGCACCAGCAGAGT |
| FTO | CTCGCATCCTCATTGGTAA | ACTTCATCTTGTCCGTTGTA |
| VEGFA | GAAGTGGTGAAGTTCATGGA | GCCTTGCAACGCGAGTCTGT |
| IGF1R | AAGTTCTGGTTGTCGAGGA | GAGCAGCTAGAAGGGAATTAC |
| COX2 | TGTCAAAACCGAGGTGTATGTA | AACGTTCCAAAATCCCTTGAAG |
