# Supplementary material for: Exploring the role of FTO in preeclampsia pathogenesis: Insights into m6A modification and decidualization
Source: Genes Dis. 2024 Dec 24;12(4):101504. doi: 10.1016/j.gendis.2024.101504 (PMC11960631; doi:10.1016/j.gendis.2024.101504)
Supplement: Multimedia component 3 [file mmc3.docx]

**Table S2. Clinical Characteristics of the Study Population**

|  | Normal pregnancy (NP, n=16) | Preeclampsia  (PE, n=16) | P value |
| --- | --- | --- | --- |
| Maternal age (years) | 32.5 ± 3.9 | 31.6 ± 4.0 | NS |
| Gestational age at birth (days) | 274.7 ± 1.8 | 250.2 ± 6.1 | <0.01 |
| Birth weight (g) | 3,370 ± 209.6 | 1,864 ± 241.8 | <0.01 |
| Systolic blood pressure (mmHg) | 118.7 ± 12.6 | 170.4 ± 15.5 | <0.01 |
| Diastolic blood pressure (mmHg) | 68.54 ± 8.7 | 112.3 ± 6.3 | <0.01 |
| Proteinuria (g/24h) | - | 7.3 ± 4.2 | <0.01 |

Data are presented as mean ± standard deviation (SD). P, Student's *t*-test.
